# Correction to: Coupled beta diversity patterns among coral reef benthic taxa

**DOI:** 10.1007/s00442-021-04886-y

**Published:** 2021-03-15

**Authors:** Jamie M. McDevitt-Irwin, Carrie Kappel, Alastair R. Harborne, Peter J. Mumby, Daniel R. Brumbaugh, Fiorenza Micheli

**Affiliations:** 1 Stanford University, Hopkins Marine Station, 120 Ocean View Blvd, Pacific Grove, CA 93950 USA; 2 National Center for Ecological Analysis and Synthesis, 735 State Street, Santa Barbara, CA 93101 USA; 3 Institute of Environment and Department of Biological Sciences, Florida International University, 3000 NE 151 Street, North Miami, Florida 33181 USA; 4 School of Biological Sciences, University of Queensland, Brisbane, St Lucia QLD 4072 Australia; 5 Department of Environmental Studies, University of California, Santa Cruz, 115 McAllister Way, Santa Cruz, CA 95060-5795 USA; 6 Elkhorn Slough National Estuarine Research Reserve, 1700 Elkhorn Road, Watsonville, CA 95076 USA; 7 Stanford Center for Ocean Solutions, 120 Ocean View Blvd, Pacific Grove, CA 93950 USA

## Correction to: Oecologia (2021) 195:225–234 10.1007/s00442-020-04826-2

Authors would like to update the missing Electronic Supplementary Material in Springerlink.

The original article has been corrected.

